# The Role of Gut Microbiota on Cholesterol Metabolism in Atherosclerosis

**DOI:** 10.3390/ijms22158074

**Published:** 2021-07-28

**Authors:** Margaret Vourakis, Gaétan Mayer, Guy Rousseau

**Affiliations:** 1 Département de Pharmacologie et Physiologie, Université de Montréal, Montréal, QC H3C 3J7, Canada; margaret.vourakis@mail.mcgill.ca; 2 Laboratory of Molecular and Cellular Biology, Montréal Heart Institute, Montréal, QC H1T 1C8, Canada; Gaetan.Mayer@icm-mhi.org; 3 Faculty of Pharmacy, Université de Montréal, Montréal, QC H3C 3J7, Canada; 4 Centre de Biomédecine, CIUSSS-NÎM/Hôpital du Sacré-Cœur, Montréal, QC H4J 1C5, Canada

**Keywords:** gut microbiota, cholesterol, atherosclerosis, hypercholesterolemia, bile acids, trimethylamine N-oxide, short-chain fatty acids, probiotics, dietary fibers, nutraceuticals

## Abstract

Hypercholesterolemia plays a causal role in the development of atherosclerosis and is one of the main risk factors for cardiovascular disease (CVD), the leading cause of death worldwide especially in developed countries. Current data show that the role of microbiota extends beyond digestion by being implicated in several metabolic and inflammatory processes linked to several diseases including CVD. Studies have reported associations between bacterial metabolites and hypercholesterolemia. However, such associations remain poorly investigated and characterized. In this review, the mechanisms of microbial derived metabolites such as primary and secondary bile acids (BAs), trimethylamine N-oxide (TMAO), and short-chain fatty acids (SCFAs) will be explored in the context of cholesterol metabolism. These metabolites play critical roles in maintaining cardiovascular health and if dysregulated can potentially contribute to CVD. They can be modulated via nutritional and pharmacological interventions such as statins, prebiotics, and probiotics. However, the mechanisms behind these interactions also remain unclear, and mechanistic insights into their impact will be provided. Therefore, the objectives of this paper are to present current knowledge on potential mechanisms whereby microbial metabolites regulate cholesterol homeostasis and to discuss the feasibility of modulating intestinal microbes and metabolites as a novel therapeutic for hypercholesterolemia.

## 1. Introduction

The gut is home to trillions of microorganisms that include fungi, parasites, viruses, and bacteria. The microbiota is a hidden organ of the human system establishing homeostasis or disease in an individual. In a healthy state, these interactions are largely symbiotic and influence a host’s nutrition, metabolism, energy, and immunity [1]. Modern methodologies have given insights into the role of the gut in many processes such as the absorption, distribution and extraction of nutrients, synthesis of vitamins, immunomodulation, and protection against pathogens [2]. Composition of the microbiota can vary between people of the same ethnicity, age, or lifestyle because there is a complex interaction between classic genetic and environmental factors. There are over 50 bacterial phyla in the gut, yet there are two predominant phyla governing the gut microbiome: the Gram-positive Firmicutes (e.g., *Enterococcus* and *Lactobacillus*) and the Gram-negative Bacteroidetes (e.g., *Bacteroides*). The other phyla are present in variable numbers and include Actinobacteria (*Bifidobacterium*), Proteobacteria, and Verrucomicrobia [3]. Overall, numerous factors such as the physiological conditions of the host (e.g., age and stress), dietary habits, environmental factors (e.g., use of medications especially antibiotic therapy), and intestinal infections can alter the diversity and composition in intestinal microorganisms creating a state of dysbiosis. This state has been associated with the pathogenesis of both intestinal and extraintestinal disorders such as irritable bowel syndrome, metabolic syndrome, obesity, diabetes, and cardiovascular system disorders [4]. Lower abundance of *Bacteroides*; higher abundance of *Lactobacillus*, *Enterobacteriaceae*, and *Streptococcus* spp.; and increased Firmicutes to Bacteroidetes ratio have been associated with cardiovascular disease (CVD) [5]. Most of these disorders may increase the permeability of the intestine, leading to increased systemic levels of bacterial products which in turn cause low-grade chronic inflammation [6]. This inflammation may promote the development of atherosclerosis and has also been hypothesized to alter plasma lipid and lipoprotein levels [7]. Despite extensive research in human and in animal models, the specific protective microorganisms depleted in dysbiotic cases and the precise molecular mechanisms causing disease remain unknown due to the complex interplay between genetic and nongenetic factors [8]. Evidence suggests that the gut microbiome is involved in the development of CVD as studies show its association with variations in body mass index and blood lipids levels, independent of age, sex, and host genetics [9].

Many findings suggest that the gut microbiota has the capacity to alter blood lipid composition in particular cholesterol, through their role in bile acid metabolism and the generation of microbial products [4,10,11,12]. The metabolism of cholesterol by gut microorganisms has been known for decades, although the specifics of these interactions on human health have only started to be considered. Given that cholesterol is an important signaling molecule in the body regulating multiple physiological functions such as BA production and hormone regulation, a disruption in its formation has many health repercussions [4]. High levels of blood cholesterol, also known as hypercholesterolemia, is a major risk factor for the development of atherosclerosis, a chronic lipid-driven inflammatory disease of the arteries, promoting myocardial infarctions and strokes [13,14]. The reduction of total cholesterol (TC) and low-density lipoprotein (LDL) cholesterol (LDLc) concentrations in the blood significantly decreases the risk of CVD [15]. Reduction of cholesterol levels can be achieved with changes to the diet and increase in physical activity, while lipid-lowering drugs are indicated only in selected situations where lifestyle modification is not possible or sufficient to reach desired target blood cholesterol concentration. It is important to note that nonpharmacological management should, as much as possible, accompany lipid-lowering therapy as it maximizes a patient’s outcome [16]. Lifestyle improvement can reduce LDLc levels by 5–15% and may lead to a meaningful CVD risk reduction [17]. Interestingly, factors influencing plasma lipids and atherosclerosis such as the consumption of dietary fatty acids, carbohydrates, and dietary fibers as well to tobacco use, obesity and level of physical activity, are also associated to changes in the gut microbial ecosystem [18].

Gut microbes are involved in the biosynthesis of an array of compounds contributing to normal human physiological functions or eliciting disease. Research over the past decade has uncovered several key microbial metabolites such as secondary bile acids, trimethylamine-N-oxide (TMAO) and short chain fatty acids (SCFAs) that may contribute to the progression and pathogenesis of atherosclerosis [19,20,21]. Although these metabolites are well studied, the processes by which they contribute to CVD remain unclear. The ability to modulate the production of these metabolites by altering the microbial composition through the diet could represent a non-pharmacological treatment for CVD [22]. The use of probiotics, prebiotics, and synbiotics has gained considerable interest as supplements for food to protect the host from enteral problems [23]. Probiotics introduce additional external microorganisms in the intestinal tract while prebiotics stimulate the growth rate of one or more of these microorganisms in the host. Both these applications can be combined (i.e., synbiotics) to improve the viability of probiotic microorganisms. Thus, the possibility to modulate lipid profiles by targeting microbial communities to control SCFA and TMAO production via dietary interventions could be used as a therapeutic tool to improve human health and decrease hypercholesterolemia. However, it is still unknown which gut microbes contribute the most to CVD and the detailed mechanisms involved require further investigation [24].

### 1.1. Cholesterol Metabolism

**Figure 1:**
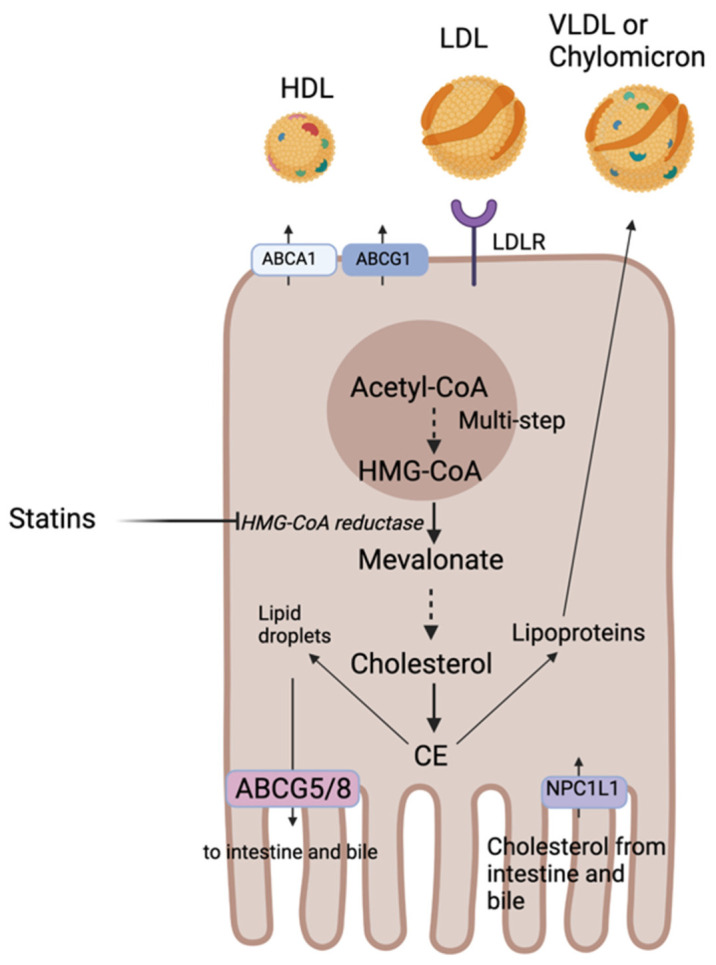
Metabolism, import, and export of cholesterol in polarized cell. The bulk of endogenous cholesterol is synthesized by the liver and starts from acetyl coenzyme A (acetyl-CoA) and concerted actions of more than 20 enzymes. Once cholesterol is formed, it is either esterified to cholesterol ester (CE) and stored in lipid droplets or secreted into the bloodstream as lipoproteins. Excess cholesterol can be excreted by ABC subfamily G member 5 and member 8 (ABCG5/8) transporters to the intestine or to the bile or packaged with lipoproteins for subsequent secretion into the blood.

Cholesterol is an essential component of eukaryotic cell membranes and the precursor of corticosteroids, sex hormones, vitamin D, and BAs. Only about one-quarter of the pooled body cholesterol is obtained from the diet mainly from animal and dairy food products, with the remaining ~75% being synthesized endogenously in the liver via the mevalonate pathway [25,26]. Cholesterol metabolism and export in the liver and intestine involve a series of enzymes and transporters (Figure 1). Dietary cholesterol can be transported into the intestine via the Niemann-Pick C1-like 1 (NPC1L1) transporter located on the apical surface of enterocytes. This transporter is also expressed on hepatocytes mediating transport of cholesterol from bile [27,28]. Circulating blood cholesterol, transported with LDL, is taken up by LDL receptors (LDLR) located on the basal surface of polarized cells. The bulk of endogenous cholesterol is synthesized by the liver and starts from acetyl coenzyme A (acetyl-CoA) and concerted actions of more than 20 enzymes. The rate-limiting enzyme of this reaction is 3-hydroxy-3-methylglutaryl coenzyme A reductase (HMGCR) and is the target of statins used for primary and secondary prevention of CVD (covered in Section 3.1) [29]. Cholesterol biosynthesis and uptake is tightly regulated by the transcription factor sterol-regulatory element-binding protein 2 (SREBP-2). SREBP-2 is embedded in the endoplasmic reticulum (ER) membrane and forms a complex with SREBP cleavage-activating protein (SCAP), also integral to the ER membrane. SCAP contains a sterol-sensing domain and when ER cholesterol exceeds 5% of total ER lipids it retains SREBP-2 in the ER in its inactive form [30]. When ER cholesterol falls below the 5% threshold, SCAP undergoes a conformational change that allows transport of SREBP-2 in COPII coated vesicles from the ER to the Golgi where two proteases sequentially cleave SREBP-2. Cleavage of SREBP-2 releases its cytosolic basic-helix-loop-helix leucine zipper domain so that it can enter the nucleus to activate transcription of all genes encoding enzymes involved in cholesterol synthesis and of the LDLR gene [31,32,33].

Once cholesterol is formed, it is either esterified to cholesterol ester (CE) and stored in lipid droplets or secreted into the bloodstream as lipoproteins. Excess cholesterol can be excreted by ABC subfamily G member 5 and member 8 (ABCG5/8) transporters to the intestine or to the bile or packaged with lipoproteins for subsequent secretion into the blood [29]. Lipoproteins carrying endogenous cholesterol are secreted by hepatocytes and are known as very low-density lipoproteins (VLDL) and the ones secreted by the intestine carry cholesterol absorbed from diet and are known as chylomicrons (covered in detail in Section 1.2). In macrophages, excess cholesterol can also be excreted via the adenosine triphosphate-binding cassette subfamily A member 1 (ABCA1) or subfamily G member 1 (ABCG1) to the blood and incorporated into the high-density lipoprotein (HDL). ABCG1 mediates the efflux of cholesterol from macrophages and thereby prevent foam cell formation and protect against atherosclerosis and CVD [34]. ABCA1 mediates the first step of reverse cholesterol transport (RCT) (further explored in Section 1.3). Mutations in ABCA1 cause Tangier disease characterized by a severe accumulation of cholesterol in macrophages responsible of prevalent atherosclerosis and premature CVD [35].

When intracellular cholesterol levels are high, liver X receptors (LXR) are activated and mediate cholesterol efflux via increased expression of the ABCG5/8 transporters [36]. Patients with mutations in genes encoding these transporters have a condition called sitosterolemia which is a disorder of sterol metabolism characterized by increased absorption and reduced biliary excretion of all neutral sterols and may exhibit hypercholesterolemia and premature heart disease [37]. The liver is the only organ capable of getting rid of excess cholesterol via its secretion into bile for conversion into BAs (further explored in Section 2.1). This is a crucial step to maintain cholesterol homeostasis in the body. Excess cholesterol decreases membrane fluidity, lipid raft signaling and generates toxic oxidative molecules such as oxysterols [33].

### 1.2. Cholesterol Transport

Because cholesterol is insoluble in water, it is packaged with triglycerides (TGs), phospholipids, and apolipoproteins (Apo) into lipoproteins that enable their transport throughout the body. There are four major classes of plasma lipoproteins—chylomicrons, VLDL, LDL, and HDL, each with unique size, density, and characteristic protein and lipid composition. They all act as lipid carriers but exert different functions throughout the body. The Apo found on their surface stabilize the complex can serve as ligands and can be cofactors for enzymes involves in the metabolism of lipoproteins [29].

#### Exogenous and Endogenous Cholesterol Pathways

**Figure 2:**
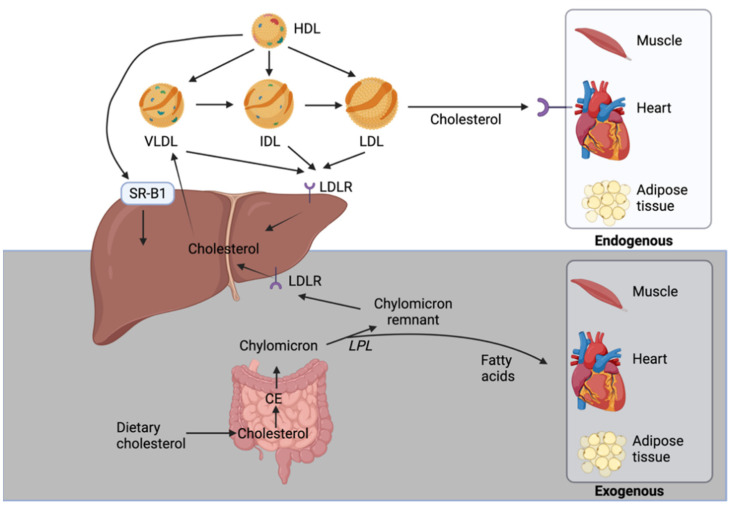
The endogenous (white) and exogenous (gray) pathways of cholesterol transport. The exogenous pathway starts with the dietary cholesterol whereas the liver is the starting point of the endogenous pathway.

In the exogenous cholesterol pathway, the dietary cholesterol is absorbed in the duodenum and proximal jejunum via NPC1L1. In enterocytes, it is esterified to CE and packaged along with apolipoproteins and TGs into chylomicrons [38]. In the circulation, the TGs of these particles are hydrolyzed by lipoprotein lipase to release fatty acids that are taken up by muscle cells and adipocytes and results in the conversion of chylomicrons to chylomicron remnants (Figure 2). The liver is responsible for removing these remnants from the circulation but smaller ones can penetrate the endothelial monolayer of an artery and participate in plaque buildup. Clinical data support the concept of chylomicron remnants increasing the risk for atherosclerosis and coronary heart disease [7].

The endogenous cholesterol pathway begins in the liver with de novo synthesis of cholesterol that is then assembled along with ApoB100, TG, and phospholipids into VLDL particles. VLDL’s TGs are broken down by lipoprotein lipase in the blood releasing fatty acids and forming intermediate density lipoproteins (IDL), which are further metabolized to cholesterol-enriched LDL (Figure 2). LDL, which carries about 70% of cholesterol in the blood, supply cholesterol to numerous tissues through LDLR-mediated endocytosis including the liver that is the predominant site of uptake. LDL can also reach the artery wall to promote atherosclerosis. Circulating LDL particles directly bind to endothelial scavenger receptor class B type 1 (SR-B1) that mediates its transcellular transport and delivery into the subendothelial space [39]. LDL particles can be trapped and oxidized in the subendothelial extracellular matrix of arteries and cause a buildup of immune cells and cholesterol forming atherosclerotic plaques [40]. Monocytes are attracted to plaques where they differentiate into macrophages, which internalize oxidized LDL particles causing them to become foam cells. Foam cells are highly inflammatory as they release chemotactic cytokines propagating a monocyte response [40]. High levels of LDL accelerate plaques progression that can ultimately block blood flow and oxygen delivery to organs such as the heart and brain. 

### 1.3. Reverse Cholesterol Transport (RCT)

**Figure 3:**
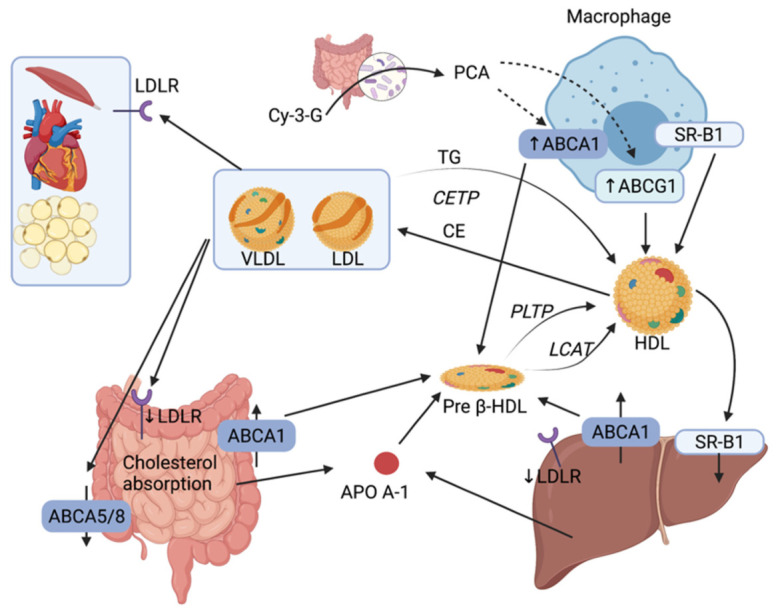
Reverse cholesterol transport pathways between intestine, liver and macrophage. RCT begins in the liver and the intestine with the synthesis of ApoA-1. ApoA-1 associates with phospholipids and free cholesterol through an interaction with ABCA1 located on various cell types (enterocytes, hepatocytes and macrophages). Gut microbiota modulates the RCT by increasing the level of ABCA1 and ABCG1 via the transformation of Cy-3-G into PCA. LDLR: LDL receptor; TG: Triglycerides; CETP: cholesteryl ester transfer protein; PLTP: phospholipid transfer protein; LCAT: lecithin-cholesterol acyltransferase; Cy-3-G: cyanidin-3-O-ß-glucoside; PCA: protocatechuic acid.

Cholesterol efflux from macrophages and its excretion by the liver is one of the most critical mechanisms for RCT. This transport plays an important role of countering toxic intracellular cholesterol accumulation and progression of atherosclerotic plaque. RCT begins in the liver and the intestine with the synthesis of ApoA-1 (Figure 3). ApoA-1 associates with phospholipids and free cholesterol through an interaction with ABCA1 located on various cell types (enterocytes, hepatocytes, and macrophages) [7]. Free cholesterol is esterified by lecithin-cholesterol acyltransferase (LCAT) and the process results in the formation of pre-β HDL particles. Pre-β HDL particles subsequently interact with scavenger receptor class B type 1 (SR-B1) and ABCG1 to incorporate more cholesterol as well as phospholipids from TG-rich lipoproteins through phospholipid transfer protein (PLTP) to form mature HDL particles. Cholesterol can then be delivered to the liver directly via hepatic SR-B1 or indirectly by transferring CE to LDL or VLDL through a process utilizing cholesteryl ester transfer protein (CETP). In addition to hepatobiliary cholesterol excretion (covered in the Section 2.1), the small intestine can also eliminate endogenous cholesterol through a pathway called the trans-intestinal cholesterol efflux (TICE) which accounts for 45% of fecal cholesterol excretion [41]. In this pathway, cholesterol enters enterocytes via LDLR and is effluxed in the intestinal lumen by ABCG5/8 transporters.

Experimental data suggest that the gut microbiota influences reverse transport by different mechanisms, including the increase in the expression of ABCA1 and ABCG1 in macrophages by a miRNA-10b-dependent mechanism involving PCA, a gut microbiota metabolite of the Cy-3-G, or by TMAO, a microbiota metabolite of L-carnitine, which inhibits the reverse transport and affect the level of circulating cholesterol (further explored in Section 2.2) [42].

## 2. Gut Bacterial Metabolites and Cholesterol Metabolism

### 2.1. Bile Acids

Cholesterol can be secreted into the bile in the unesterified form via the ABCG5/G8 heterodimer or converted into BAs in the liver. BA excretion is the major pathway for cholesterol export from the body, apart from neutral sterol excretion, and plays a key role in cholesterol, triglycerides, glucose, and energy homeostasis through receptor mediated processes [4,43]. It is also crucial for absorption of dietary fats and lipid-soluble vitamins [44]. Bacteria in the lower intestine can process cholesterol to coprostanol and the latter, unlike cholesterol, is poorly absorbed by the intestinal mucosa as it is the most abundant sterol in the feces [45]. Notably, a high efficiency of cholesterol to coprostanol metabolism was suggested to reduce CVD risk [4]. The main bacteria taxa carrying this conversion involve *Lactobacillus* and *Eubacterium* (*Eubacterium coprostanoligenes*), however bacterial enzymes responsible for this conversion remain unknown [4].

**Figure 4:**
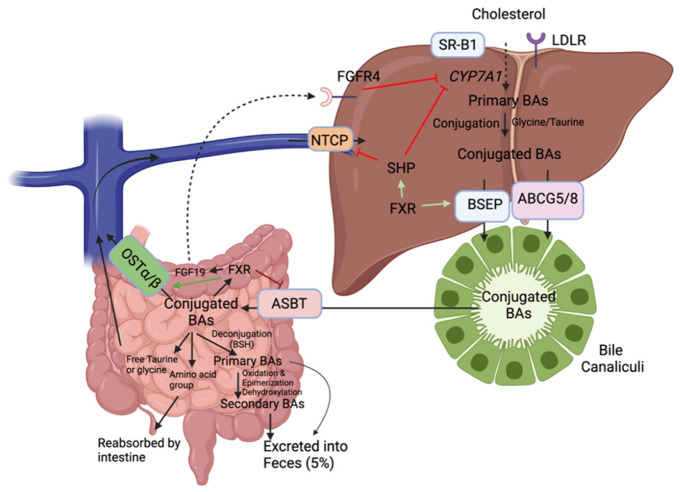
Enterohepatic recirculation of bile acids via reverse cholesterol transport. In the liver, cholesterol is metabolized to BAs by hepatic cytochromes, notably 90% of the time by the rate-limiting enzyme cholesterol 7 α-hydroxylase (CYP7A1). When BAs are conjugated, they are actively transported into bile via the bile salt export pump (BSEP) or via ABCG5/8. In the small intestine, certain gut microbes contain an enzyme known as bile salt hydrolase (BSH) capable of deconjugating (i.e., remove glycine or taurine conjugates) BAs preventing their reuptake by the ASBT transporter BAs: Bile acids; apical sodium-dependent bile acid transporter: ASBT; organic solute transporters: OST α/β; nuclear farnesoid X receptor: FXR; bile salt export pump: BSEP; Na+/taurochlorate cotransporting polypeptide: NTCP; fibroblast growth factor receptor 4 (FGFR4).

In the liver, cholesterol is metabolized to BAs by hepatic cytochromes, notably 90% of the time by the rate-limiting enzyme cholesterol 7 α-hydroxylase (CYP7A1) (classical pathway) (Figure 4). When the BA pool (i.e., total amount of BAs present in the enterohepatic circulation) increases, nuclear farnesoid X receptor (FXR) induces the expression of small heterodimer partner (SHP), which in turn represses CYP7A1expression, the rate-limiting enzyme of BA synthesis [46]. The remaining 10% of the time, cholesterol is processed by sterol 27-hydrolyase (CYP27A1) in extrahepatic sites including the vascular endothelium and macrophages (alternative pathway). The initial products are cholic acid and chenodeoxycholic acid (primary BAs) which are then conjugated with the amino acids glycine (mainly in humans) or taurine (mainly in rodents) for excretion into bile and further passage in the intestine [47]. These conjugated BAs are actively transported into bile via the bile salt export pump (BSEP) or via ABCG5/8. They can be stored temporarily as mixed micelles along with cholesterol and phospholipids in the gallbladder to be released into the duodenum upon meals to facilitate emulsion.

Approximately 95% of BAs are reabsorbed from the intestine, predominantly as conjugated BAs, mainly via the apical sodium-dependent bile acid transporter (ASBT) and a small amount can also enter the hepatocyte via passive diffusion (Figure 4) [48]. Then, they are secreted into the portal circulation by the organic solute transporters (OST α/β) to be transported back to the liver. In the ileum, BAs can activate FXR, which inhibits ASBT, induces OST α/β and the production and secretion of fibroblast growth factor 19 (FGF19) in the portal circulation. This factor will bind to the fibroblast growth factor receptor 4 (FGFR4) and activate a signaling pathway that will repress CYP7A1 transcription [49]. On the basolateral membrane of hepatocytes, Na+/taurochlorate cotransporting polypeptide (NTCP) mediates the uptake of conjugated BAs and organic anion transporting proteins that of unconjugated BAs. This process is called enterohepatic circulation and it occurs about six times a day in humans [47]. It is tightly negatively regulated by the FXR receptor both in the liver and intestine [46]. Not only can this receptor inhibit hepatic CYP7A1, but it can also regulate basolateral uptake by stimulating BSEP expression and repressing NTCP expression via SHP to prevent intrahepatic BA accumulation [44].

In the small intestine, certain gut microbes contain an enzyme known as bile salt hydrolase (BSH) capable of deconjugating (i.e., remove glycine or taurine conjugates) BAs preventing their reuptake by the ASBT transporter (Figure 4). BSH genes have been detected in Gram-positive gut bacteria including *Lactobacillus*, *Clostridium*, *Listeria*, and *Bifidobacterium*. Members of the *Bacteroides* genus are the only Gram-negative bacteria with BSH activity [4]. This deconjugation produces amino acid groups and free taurine or glycine which are reabsorbed into the intestine and return to the liver, respectively. It also produces free cholic acids (unconjugated bile salts) that are less efficiently reabsorbed which results in their excretion into the feces. Primary BAs can be further metabolized through gut microbial 7α-dehydroxylation to secondary BA known as lithocholic acid (from chenodeoxycholic acid) and deoxycholic acid (from cholic acid) [47]. The known bacterial species that possess 7α-dehydroxylation activity are members of the Firmicutes phylum (*Clostridium* and *Eubacterium*) [4]. Quantitatively, these members possess the most important enzymatic activity as secondary BAs predominate in the feces [44].

Secondary BAs have often been discussed as having negative impact on human health and their increased concentration in the intestine may promote the development of carcinomas [50]. Primary and secondary BAs compose the total BA pool, in a relatively fixed proportion [44]. If the ratio of bile salts to cholesterol changes, cholesterol can precipitate with calcium salts and bile pigments and may drive diseases such as gallstone disease and non-alcoholic fatty liver diseases [51,52]. Furthermore, serum levels of primary BA were reduced and specific secondary BAs increased in patient with chronic heart failure [53]. Microbial metabolism leads to a more hydrophobic BA pool facilitating excretion into feces of the 5% remaining BAs.

### 2.2. Trimethylamine (TMA) and Trimethylamine-N-Oxide (TMAO)

**Figure 5:**
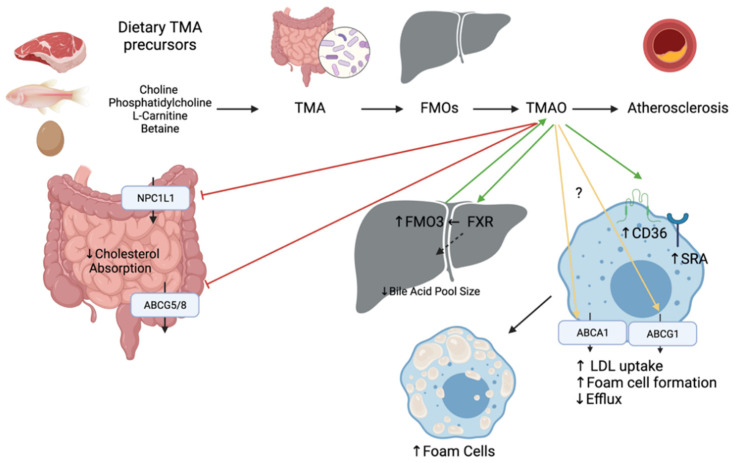
Mechanisms linking the pro-atherosclerotic activity of gut microbial metabolite TMAO and cholesterol transport and synthesis in the intestine, liver, and macrophage. The intestinal microbiota metabolizes choline, phosphatidylcholine, L-carnitine, and betaine to trimethylamine (TMA), which is oxidized into TMAO by hepatic flavin monooxygenases (FMO). Green arrows indicate an increase of expression or activation whereas red arrows indicate a reduction of the expression. Yellow arrows indicate that the results in the literature are controversial.

The intestinal microbiota metabolizes choline, phosphatidylcholine, L-carnitine, and betaine to trimethylamine (TMA), which is oxidized into TMAO by hepatic flavin monooxygenases (FMO3) (Figure 5). Foods of animal origin such as dairy products, eggs, and red meat are all rich in TMA precursors, thus a potential source of TMAO. TMA and TMAO can also be acquired from fish and other seafood [54]. When fish were compared to other foods rich in carnitine and choline, fish have the highest levels of TMAO and marked metabolites [54]. TMAO levels in serum are dictated by genetic variations, gut microbiota, and the diet [55]. Elevated blood TMAO levels have been linked to poor outcomes in patients with CVDs such as coronary heart disease and chronic heart failure and can be a valuable prognostic factor for adverse cardiac events for patients with chronic heart failure after myocardial infarction [19,56]. Inhibition of gut microbiota-dependent TMAO production has been shown a promising strategy for the treatment of atherosclerosis [57].

The underlying mechanisms whereby TMAO contributes to CVD are not fully elucidated, yet mechanisms related to cholesterol metabolism have been proposed (Figure 5). A study performed with ApoE^−/−^ mice fed TMAO for 8 weeks demonstrated accelerated aortic lesion formation. Mechanistically, it downregulates CYP7A1 and CYP27A1 enzymes involved in the BA synthesis, which might be mediated by the activation of SHP and FXR [58]. The activation of FXR inhibits cholesterol absorption by modulating BA pool size and composition, thus leading to decreased RCT [46]. Injection of FXR has been shown to induce the expression of FMO3 and the production of TMAO in mice. Dietary supplementation of TMAO was also shown to reduce expression of both intestinal transporters NPC1L1 and ABCG5/8 in vivo [59]. However, another study has shown that TMAO caused an upregulation of ABCG5/8 in the small intestine in rats fed a high fat high cholesterol diet [60]. Expression of ABCA1 and ABCG1 in macrophages also seems contradictory as some studies show no differences and others show a downregulation upon TMAO treatment [59,60,61]. Last, TMAO is thought to upregulate cluster of differentiation 36 (CD36) and scavenger receptor A (SRA) located on macrophages and involved in the uptake of oxidized LDL [62]. This enhances the formation of foam cells which are the most critical component directly related to atherosclerosis.

There is strong evidence demonstrating that TMAO is not only an independent risk factor for CVD, but also a therapeutic target for CVD on the basis of a large amount of experimental and clinical data [63]. TMA and FMO3 are also important targets to consider as they participate in the formation of TMAO, but they have also shown clinical evidence of disrupting cholesterol homeostasis. Inhibition of TMA lyase has been shown to increase loss of fecal neutral sterol in the form of coprostanol and alter the total amount of composition of circulating bile acids [64]. This inhibition was achieved with small molecule drugs directly targeting TMA lyase which have previously shown potent anti-atherothrombotic activity and hold therapeutic potential for diverse cardiometabolic disorders [4]. In contrast to the microbiota’s cholesterol-lowering effects by coprostanol generation and the formation of secondary bile acids, the causative role of TMAO in atherogenesis is under debate [65]. FMO3 knockdown has been shown to alter biliary excretion, intestinal absorption and limit the production of hepatic oxysterols and cholesteryl esters in cholesterol-fed mice [66]. Thus, these dietary metabolites represent markers mediating disease and are an interesting avenue to further explore treatments for CVD risk reduction.

### 2.3. Short-Chain Fatty Acids

SCFAs are crucial for intestinal health as they mediate the interaction between the diet, the gut, and the host. In doing so, they are involved in multiple metabolic processes such as the synthesis of lipids, fat storage, glucose uptake, and inflammation [20]. Once dietary fibers and resistant starches enter the colon, they are fermented and transformed to SCFAs such as butyrate, acetate, and propionate which account for ~90% of total SCFAs in the human colon [20]. Their production depends on the microflora present in the colon as well as the substrate source (diet), environment condition including pH and the gut transit time [67]. The main bacteria producing SCFAs are the clostridial clusters IV and XIVa of Firmicutes including species of *Eubacterium*, *Roseburia*, *Faecalibacterium*, and *Coprococcus* [44]. Hundreds of gut bacterial species across many taxa share the genes for fermenting carbohydrates into SCFAs [68]. Acetate and propionate are mainly produced by Bacteroidetes, whereas Firmicutes are the primary contributors of butyrate [69]. The concentrations of SCFAs changes along the GI tract with the highest concentrations in the proximal colon and cecum where they can be used locally by the enterocytes or transported across the epithelium into the bloodstream [20]. Between 90% to 99% of SCFAs are absorbed in the gut or used by the microbiota [20]. High SCFA concentration in the gut lumen can lower colonic pH and impact microbial communities inhibiting the growth of potential pathogens including *Salmonella* spp. and *Escherichia coli* and promote the growth of beneficial bacteria such as *Lactobacilli* and *Bifidobacteria*, which are known to reduce the risk of CVDs [48,70,71].

**Figure 6:**
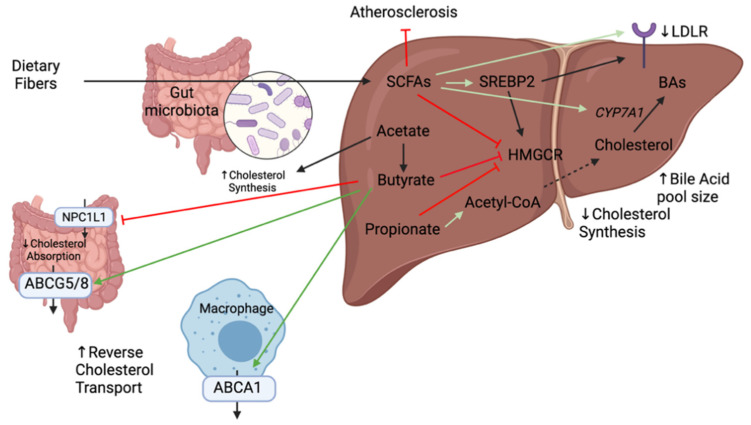
Mechanisms linking the anti-atherosclerotic activity of short-chain fatty acids (SCFAs) and cholesterol transport and synthesis in the intestine and liver. Dietary fibers could be fermented and transformed into short chain fatty acids (SCFA) by some species present in the gut. Green arrows indicate an increase of activity or expression whereas the red arrows indicate an inhibition or reduction of expression.

Numerous mechanisms that link dietary fibers to the reduction of cholesterol levels have been elucidated notably through the production of SCFAs (Figure 6). SCFAs have been shown to lower cholesterol synthesis rate thereby reducing cholesterol in the serum [70]. The addition of SCFAs in the diet of hamsters on a high-cholesterol diet showed an upregulation of SREBP2, LDLR, and CYP7A1 genes in the liver thereby promoting hepatic uptake of serum cholesterol and fecal excretion of BAs [72]. The cholesterol lowering properties of SCFAs can be further extended to individual SCFAs. Propionate has been shown to inhibit the incorporation of acetate into fatty acids and cholesterol in rat hepatocytes thereby decreasing cholesterol serum levels [71]. It can also stimulate the hepatic synthesis of bile salts by upregulating CYP7A1 [73]. Acetate, the most abundant SCFA in peripheral circulation, is a substrate for cholesterol and, therefore, promotes cholesterol synthesis [71]. Therefore, a decrease in the acetate to propionate ratio may result in a decrease in serum lipids which is an interesting avenue to decrease cardiovascular risk. It can also be transformed into butyrate and participate in long-chain fatty acid synthesis [20]. Butyrate is the most beneficial in terms of colonic health as it is the key energy source for colonocytes and enterocytes and promotes normal cell differentiation and proliferation [74]. It also has numerous beneficial health effects as it protects from pathogen invasion, modulates the immune system and reduces cancer progression. It is locally consumed, where other absorbed SCFAs drain into the portal vein [74]. It has been shown to inhibit the HMGCR activity, thus preventing cholesterol synthesis [70]. As SCFAs levels are usually low in the circulation, local effects of butyrate were evaluated on Caco-2 cells (intestinal permeability model). Butyrate was shown to suppress cholesterol uptake in a dose-dependent fashion by inhibiting NPC1L1 and increasing expression of ABCG5/8 [75]. It was also shown to protect against high-fat induced atherosclerosis by upregulating ABCA1 in ApoE^−/−^ mice [76]. At present, oral butyrate is commonly used as a supplement but has low bioavailability and most butyrate producing bacteria are anaerobic which limits the production capacity of this metabolite. Studies are being done to evaluate butyrate concentration in the small intestine in order to develop oral formulations that maintain pharmacologically active butyrate concentrations [77]. Collectively, several studies point towards an important role of SCFAs in cholesterol levels. However, there are contradicting results on their mechanisms of action and further studies demonstrating solid evidence are crucial to move into human studies.

## 3. Cholesterol-Lowering Agents

Recently, evidence indicated that gut microbiota can be modified by drugs and by nutritional interventions. Interactions between statins and gut microbiota will be explored to determine the involvement of the gut microbiota in their beneficial effects. After that, probiotics will be further investigated as a potential therapy to reduce the levels of cholesterol.

### 3.1. Statins

Statin therapy represents the privileged method to achieve therapeutic circulating LDLc targets in patients for both primary and secondary prevention of CVD. It is one of the most potent treatments to decrease LDLc levels and, as per current guidelines, first-line therapy for atherosclerotic CVD. Statins inhibit HMGCR which is the rate-limiting enzyme of the mevalonate pathway that forms cholesterol [33]. As a result, cholesterol synthesis is inhibited in the liver leading to the activation of SREBP2 and upregulation of LDLR on the membrane of hepatocytes. This ultimately leads to an increased uptake of LDLc by the liver and lower circulating cholesterol levels [78]. Together with age, sex, blood pressure, diabetes and smoking, and total cholesterol, LDLc and HDLc levels are important risk factors used to calculate the Framingham Risk Score (FRS; Canadian guidelines) or the clinician–patient risk discussion (ACC/AHA guidelines) that will determine if a statin will be recommended to a patient for primary prevention of CVD. The higher the ratio of LDLc (or total cholesterol)/HDLc, the higher the probability that a statin will be recommended as LDLc is directly associated with CVD while HDLc is inversely associated with CVD [79,80]. The effects of statins are pleiotropic meaning they go beyond cholesterol reduction. They exhibit anti-inflammatory, immunomodulatory and antithrombotic actions [81]. They have also been reported to alter the gut microbiome as their use has been associated with decreased TMAO [82]. However, statins present many limitations. They present increased risk of diabetes, myopathy and hepatic damage [78]. Furthermore, treatment response is individual and varied. A meta-analysis evaluated statin interventional trials encompassing 32,258 patients from 37 trials and found that 5.3% to 53.3% percent of patients experience a suboptimal response (less than 30% reduction in LDLc levels). This study concluded that there is high interindividual variation in LDLc reduction at all doses of simvastatin, atorvastatin, and rosuvastatin [83]. Other factors such as smoking, diet, exercise, and genetic polymorphisms can affect LDLc levels and, thus, statin doses must be adjusted. Even when doses are adjusted, suboptimal adherence to the therapy represents another challenge [84]. As statin therapy exhibits potential antibacterial activities, the gut microbiota may modulate the therapeutic actions of these drugs [85].

Hyperlipidemic patients often display intestinal disorders that may further impair lipid metabolism [86]. An in vivo study was conducted on 64 hyperlipidemic patients treated with rosuvastatin for 4 to 8 weeks and investigated the role of the microflora in mediating the lipid-reduction efficacy of rosuvastatin. They found variations in the community composition, taxa, and diversity of the gut microbiota in association to the LDLc-lowering response of the statin [87]. These results indicate that it is possible to modulate the gut microflora for a better statin response. A study conducted by Sun et al. assessed bacterial composition and diversity of 202 hyperlipidemic patients with either statin-sensitive or statin-resistant response. The results indicated that statin-sensitive patients exhibited a higher gut biodiversity, and this group showed an increased genera of *Lactobacillus*, *Eubacterium*, *Faecalibacterium*, and *Bifidobacterium* and decreased genus of *Clostridium* when compared to the statin-resistant group [88].

Participants with a poor treatment response to rosuvastatin also showed a significant increase in TMAO values compared to other participants treated with placebo [89]. Another study evaluated gut microbial communities in hypercholesterolemic patients treated with atorvastatin. When compared with healthy and untreated subjects, statin-treated patients exhibited an increased abundance of anti-inflammation-associated bacteria (*Faecalibacterium prausnitzii* and *Akkermansia muciniphila*) and a decreased abundance of bile-associated species (*Bifidobacterium bifidum*) [90]. Thus, defining the gut microbiota diversity and metabolites could be used as a valuable tool to predict a patient’s statin response and optimize statin dosages in personalized CVD treatment regimens. Further research is needed to determine whether these changes in the gut are directly caused by statins or if alterations in the microbiota are a consequence of host response to these statins.

### 3.2. Probiotics

Because of the high cost and deleterious side effects of many pharmacological agents, recent interest has sparked for probiotics therapies as a convenient approach to lower serum cholesterol levels. Probiotics are currently defined by the World Health Organization as « live microorganisms that, when administered in adequate quantities, confer health benefits to the host » [91]. They present a cheap, noninvasive approach with minimal to no side effects to reduce coexisting CVD risk factors such as TC, LDLc, weight, waist, and inflammation markers [92]. Probiotics strains must meet safety and functionality criteria and maintain their properties through distribution and storage. Some required properties include human or animal origin, non-pathogenic, isolated from a healthy GI tract, resist bile salts and enzymes and be able to antagonize pathogens such as *Clostridium difficile* and *Helicobacter pylori* [23]. Probiotics can be found in fermented foods of vegetable origin such as sauerkraut and pickles, and may also be added to unfermented foods like milk, juices, and cereals [93]. Their benefits are specific to species and strains and thus cannot be extrapolated to other strains without confirmation [94].

A meta-analysis of 30 randomized controlled trials found that 1624 participants treated with probiotics showed a reduction of TC and LDLc compared to control subjects by 7.8 mg/dL and 7.3 mg/dL, respectively. No change was observed for levels of HDL or TGs [95]. A larger meta-analysis incorporating 32 randomized controlled trials, showed that the probiotic group had lower serum TC levels than the control group with a mean difference (MD) of −13.27 mg/dL [96]. Another meta-analysis by Shimizu et al. including 11 randomized controlled trials, found that probiotic supplementation could be useful in the treatment of hypercholesterolemia as they observed a reduction of TC (MD −0.17 mmol/L) and LDLc (MD −0.22 mmol/L) [97]. As these meta-analyses had many limitations, the authors concluded that the existing clinical evidence was not strong enough to endorse probiotics as an alternative therapy to improve blood lipid profiles [95,96,97]. A review assessed probiotic supplementation in healthy adults and found 14 studies on lipid profiles and cardiovascular risk. Authors determined that there was insufficient evidence to support the role of probiotics in improving blood lipid profiles [98]. The most well-studied human probiotic microorganisms for their cholesterol-lowering effects in humans and animals belong mostly to *Lactobacillus* and *Bifidobacterium* genera [99]. Based on meta-analysis studies, specific probiotic strains that reduced TC in patients are *B. lactis* and *L. acidophilus* (MD of −8.30 mg/dL), VSL #3 (MD −11.04 mg/dL), and *L. plantarum* (MD −1.56 mg/dL) [96]. Consuming *Lactobacillus* significantly reduced TC by 0.26 mmol/L and LDLc by 0.23 mmol/L. Among probiotic *Lactobacillus* genus, sub-analyses showed the greatest reduction of TC was observed with *L. plantarum* and the greatest reduction of LDLc was with *L. plantarum* and *L. reuteri* [100]. Another study demonstrated that *L. acidophilus* strain has been shown to cause the greatest TC and LDLc reductions (MD −0.35 mmol/L) in normal to mildly hypercholesterolemic individuals [97,101].

#### 3.2.1. Combination of Probiotics

Recently, studies have tried combining multiple strains of probiotics to increase their hypocholesterolemic action. In a study conducted by Kim et al. low, medium and high doses of a probiotic mixture containing two *Lactobacilli* (*L. reuteri* and *L. plantarum*) and three *Bifidobacteria* (*B. longum*, *B. lactis*, and *B. breve*) strains were given to hypercholesterolemic rats [102]. These treated groups, compared to the control group not given probiotics, experienced a TC reduction by 1.2-fold, 1.5-fold, and 1.3-fold, respectively. TG levels were also decreased by 1.32-fold, 1.4-fold, and 1.4-fold in low, medium, and high doses of probiotic-treated groups when compared to the control. A similar reduction (1.3-fold, 1.4-fold, and 1.5-fold in low, medium, and high doses of probiotic-treated groups, respectively) was observed for LDLc [102]. A multi-strain probiotic mixture VSL #3 containing 4 strains of *Lactobacilli* (including *L. plantarum*)*,* 3 strains of *Bifidobacteria* and *Streptococcus thermophilus* showed reduced inflammation and atherosclerotic plaque development in ApoE^−/−^ mice [103]. In humans, a combination of two most popular probiotics (*L. acidophilus* and *B. bifidum)* showed decreased serum TC (MD −40.1 mg/dL) and LDLc (MD −28.2 mg/dL) levels in hypercholesterolemic patients treated over a six-week period [101,104].

#### 3.2.2. Probiotics and TMAO

Research concerning the impact of probiotic supplementation on decreasing serum TMAO levels is limited. Probiotics could modulate the microbiome in a way to inhibit pathogenic strains responsible of TMAO synthesis. In female BALBc mice, diet supplementation with *Enterobacter aerogenes* (ZDY01) showed a 53% reduction in serum TMAO, which could be explained by the reduction in cecal TMA [105]. *Lactobacillus plantarum* (ZDY04) was able to reduce serum TMAO levels via the modulation of gut microbiota, increasing abundance of *Lachnospiraceae* by 78.8% and *Bacteroidaceae* from 1.84% ± 2.55% to 3.56% ± 3.47% and decreasing the relative abundance of *Mucispirillum* genus by 67%. Furthermore, it mediated an atheroprotective effect in ApoE^−/−^ mice by decreasing atherosclerotic lesion size observed in aortic sinus sections and whole aortas [106]. *Enterococcus faecium* (WEFA23) was also shown to improve the diversity of the gut microbiota in rats fed a high-fat diet and decreased TMAO and cholesterol levels [107]. This probiotic showed enhanced synthesis of BAs via CYP7A1 upregulation, increased the expression of LDLR involved in cholesterol transport and downregulated the expression of HMGCR involved in cholesterol synthesis.

#### 3.2.3. Suggested Hypocholesterolemic Mechanisms of Probiotics

**Figure 7:**
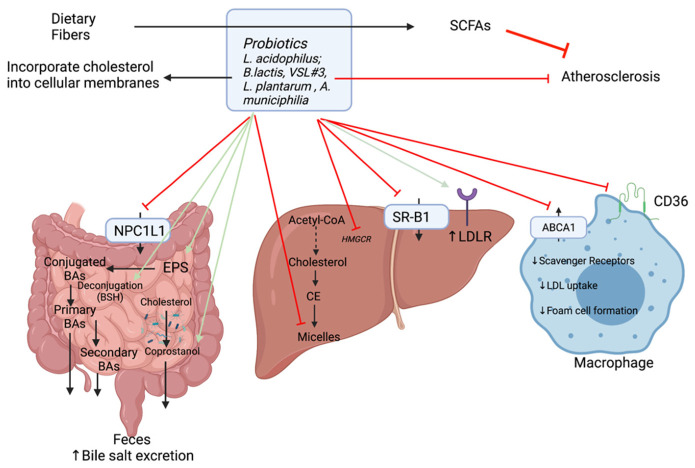
Hypocholesterolemic mechanisms of action of probiotics. It has been observed that some probiotics could reduce cholesterol and ultimately atherosclerosis. Some of the major strains used as probiotics are indicated. The mechanisms are still speculative and could be associated with an increase (green arrows) or a decrease (red arrows) of the expression or activities.

Based on in vitro and animal experiments, there are several possible mechanisms for the removal of cholesterol (Figure 7):

(1) Some probiotics are thought to reduce the enterohepatic circulation of bile salts through the regulation of BSH activity (Section 2.1). As the excretion of deconjugated BAs increases, there is a greater need to mobilize systemic cholesterol to the liver for *de novo* BA production. This provokes an increase in LDLR expression and hepatic uptake of LDLc from the circulation. On this account, serum LDLc (1.28 mM vs. 2.46 mM) and TC concentrations (3.49 mM vs. 4.47mM) are reduced in mice given probiotics [73,108]. Moreover, with increased BSH activity, there are fewer BAs to participate in micelle formation. Micelles play a role in the absorption of cholesterol in the intestine and are composed of cholesterol, bile salts, and phospholipids. The cholesterol assimilation abilities of selected strains of *Lactobacillus* were shown to vary from one strain to another. For example, in in vitro experiments *L. acidophilus* KU41 and M23 were found to reduce cholesterol levels by 50% while *L. casei* MB3 decreased cholesterol slightly below 30%. Thus, these strains were able to remove cholesterol by inhibiting micelle formation [109].
(2) Another mechanism whereby probiotics reduce cholesterolemia is the incorporation of cholesterol into bacterial cellular membranes [110,111]. Cholesterol assimilation was significantly higher in growing cells than resting and dead cells. Both live and heat-killed *Lactococcus lactis subsp. lactis N7* cells were able to remove cholesterol from growth media [111].
(3) Subsequently, the gut microbiota plays a role on cholesterol conversion into coprostanol making it easily eliminable with defection and reducing its absorption [4]. The efficiency of this conversion depends on the abundance of cholesterol-reducing bacteria (possessing reductase enzyme). This enzyme is found in some strains of probiotic bacteria such as *L. acidophilus* ATCC 314, *L. acidophilus* FTCC 0291, *L. bulgaricus* FTCC 0411, and *B. bifidum* PRL2010 [73].
(4) Probiotic bacteria can modulate key cholesterol transport by down-regulating gene expression of NPC1L1, ABCA1, CD36, and SR-B1 [112,113].
(5) They can also modulate cholesterol synthesis by inhibiting HMGCR notably through the production of SCFAs allowing a redistribution of cholesterol from the plasma to the liver [20].
(6) In addition to the production of SCFAs, gut microorganisms notably lactic acid bacteria (LAB) (including *Lactobacillus* and *Bifidobacterium* species) can synthesize different polysaccharides such as exopolysaccharides (EPS). EPS are extracellular polysaccharides secreted or attached to the bacterial cell wall which can affect adhesion and provide protection from environmental stresses. In addition, they have the potential to act as prebiotics as they can affect the GI microbiome and lower serum cholesterol levels [70]. There is a correlation between the amount of EPS produced and the quantity of cholesterol assimilated by the strain. The EPS produced by *Lb. plantarum* BR2 and *L. paracasei* M7 exhibited cholesterol-lowering properties [114]. The proposed mechanism for this action is the binding to free BAs, which thereby increases their excretion via the feces.

These mechanisms are hypothetical and further studies are needed to elucidate which ones play dominant roles.

## 4. Conclusions

This study provides a general understanding of cholesterol transport and metabolism in the body and deregulated mechanisms when cholesterol homeostasis is disrupted. Lowering TC and LDLc plasma levels during hypercholesterolemia is extremely useful for diminishing the risk of CVD. It also highlights potential nutritional interventions that may restore microbial populations and can be used as an adjuvant to traditional treatments for hypercholesteremia. According to the literature at this time, we believe the first of line treatment to restoring a healthy gut microbiota should be lifestyle interventions such as improving dietary habits and achieving exercise recommendations. A greater understanding of the host–microbiota–metabolite interactions may promote a shift towards the development of customized nutritional plans and probiotics that are specifically designed according to bacterial deficiencies. Statin therapy has prescribed and evidence-based lipid-lowering drug for lowering LDLc but in higher doses, is associated with an increased risk of hepatic and muscular side effects (as discussed in Section 3.1). A subset of patients on statins cannot achieve target plasma cholesterol levels even at the highest doses and decide to stop treatment altogether than deal with the side effects [115]. Co-therapy involving a statin in combination with a probiotic is another interesting avenue to explore that can exert a combine cholesterol-lowering effect thereby decreasing statin doses [116]. However, it is presently very difficult to utilize knowledge of microbiota composition and function in clinical practice. There are no outcome studies proving that nutraceuticals can prevent CVD morbidity or mortality. Many of the proposed mechanisms and experimental evidence targeting cholesterol-lowering effects remain controversial and have never been directly observed in humans. Furthermore, there exists a gap in the knowledge about the impact of gut microbiota on CVD. Attempts to establish a common “human core microbiome” are challenging due to strong influence of genetic and environmental factors. As research on the gut increases, the correlation of some bacterial populations and different physiological states strengthens and molecular mechanisms contributing to disease are slowly being discerned. With the current advances in technology, metabolic profiling and others “omic” analytic platforms can be useful tools to discover candidate microbial communities and their derived metabolites that impact human health [117]. Such advances will help discern the gut microbial mechanisms that explain the relationship between intestinal microbiota with cholesterol metabolism. This will lead to deeper understanding of the microbiota and encourage the development of microbiota-based therapies and microbiome-informed precision medicine in order to reduce the global prevalence of CVD.
